# Supplementary material for: Cholesterol Metabolite 27-Hydroxycholesterol Enhances the Secretion of Cancer Promoting Extracellular Vesicles by a Mitochondrial ROS-Induced Impairment of Lysosomal Function
Source: bioRxiv. 2024 May 1:2024.05.01.591500. Preprint. [Version 1] doi: 10.1101/2024.05.01.591500 (PMC11092642; doi:10.1101/2024.05.01.591500)

Title: Cholesterol Metabolite 27-Hydroxycholesterol Enhances the Secretion of Cancer Promoting Extracellular Vesicles by a Mitochondrial ROS-Induced Impairment of Lysosomal Function

Authors: Anasuya Das Gupta[1], Jaena Park[2,3], Janet E. Sorrells[2,3], Hannah Kim[1], Natalia Krawczynska[1,3], Hashni Epa Vidana Gamage[1], Adam T. Nelczyk[1], Stephen A. Boppart[2, 3, 4, 5, 6, 7, 11], Marni D. Boppart[3, 5, 8], and Erik R. Nelson[1, 3, , 9, 10, 11, *]

[1] Department of Molecular and Integrative Physiology, University of Illinois at Urbana-Champaign, Urbana Illinois, 61801 USA.
[2] Department of Bioengineering, University of Illinois Urbana-Champaign, Urbana, Illinois, 61801, USA.
[3] Beckman Institute for Advanced Science and Technology, University of Illinois at Urbana-Champaign, Urbana Illinois, 61801 USA.
[4] Department of Electrical and Computer Engineering, University of Illinois Urbana-Champaign, Urbana Illinois, 61801 USA.
[5] Carle Illinois College of Medicine, University of Illinois Urbana-Champaign, Urbana Illinois, 61801 USA.
[6] Interdisciplinary Health Sciences Institute, University of Illinois Urbana-Champaign, Urbana Illinois, 61801 USA.
[7] NIH/NIBIB Center for Label-free Imaging and Multi-scale Biophotonics (CLIMB), University of Illinois Urbana-Champaign, Urbana, Illinois, 61801 USA.
[8] Department of Kinesiology and Community Health, University of Illinois Urbana-Champaign, Urbana Illinois, 61801 USA.
[9] Carl R. Woese Institute for Genomic Biology- Anticancer Discovery from Pets to People, University of Illinois at Urbana-Champaign, Urbana Illinois, 61801 USA.
[10] Division of Nutritional Sciences, University of Illinois Urbana-Champaign, University of Illinois at Urbana-Champaign, Urbana Illinois, 61801 USA.
[11] Cancer Center at Illinois, University of Illinois Urbana-Champaign, Urbana Illinois, 61801 USA.

[*] Corresponding author. Please address all correspondence to Erik R. Nelson. Email: enels@illinois.edu

Supplementary Figures

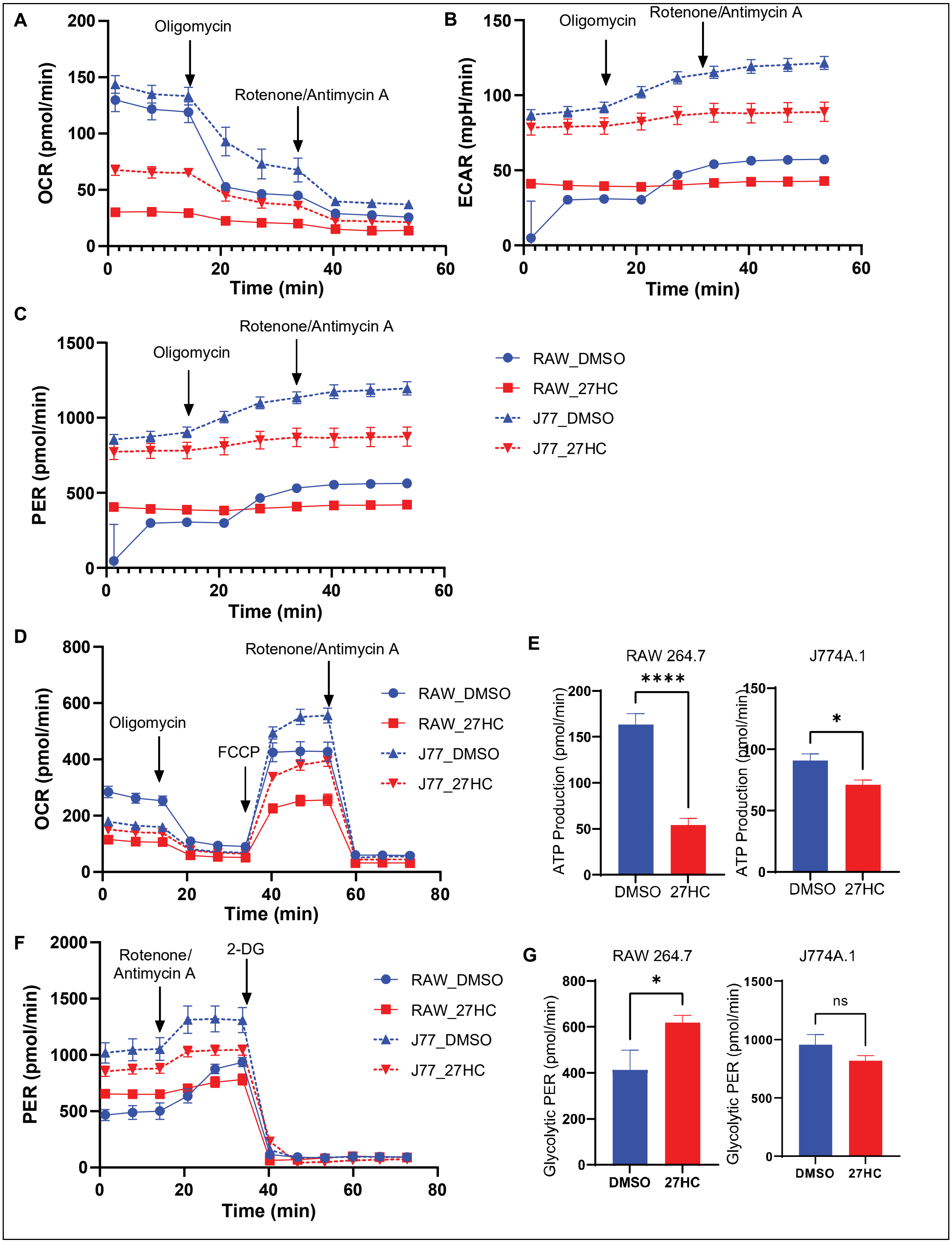

Supplement: Supplement 1 — Supplementary Figure 1: (A) Seahorse ATP Production Assay indicating oxygen consumption rate (OCR), (B) extracellular acidification rate (ECAR) and (C) PER rates (n=6). (D) Seahorse Mito Stress Test indicating OCR and (E) Mitochondrial ATP production rate (n=6). (F) Seahorse Glycolysis Stress Test indicating PER and (G) glycolytic PER (n=5/6). Statistical analyses were performed using a Student’s t-test ****P-value<0.0001, *P-value<0.05. Data are presented as mean+/−SEM. [file media-1.pdf]
